# Reconstruction of a Genome-Scale Metabolic Model of *Streptomyces albus* J1074: Improved Engineering Strategies in Natural Product Synthesis

**DOI:** 10.3390/metabo11050304

**Published:** 2021-05-11

**Authors:** Cheewin Kittikunapong, Suhui Ye, Patricia Magadán-Corpas, Álvaro Pérez-Valero, Claudio J. Villar, Felipe Lombó, Eduard J. Kerkhoven

**Affiliations:** 1 Department of Biology and Biological Engineering, Chalmers University of Technology, 41296 Gothenburg, Sweden; cheewin@chalmers.se; 2 Department of Functional Biology, IUOPA (Instituto Universitario de Oncología del Principado de Asturias) and ISPA (Instituto de Investigación Sanitaria del Principado de Asturias), University of Oviedo, 33006 Oviedo, Spain; yesuhui@uniovi.es (S.Y.); patricia.macor@gmail.com (P.M.-C.); apv.moratalla@gmail.com (Á.P.-V.); cjvg@uniovi.es (C.J.V.); lombofelipe@uniovi.es (F.L.)

**Keywords:** genome-scale metabolic model, *Streptomyces albus* J1074, heterologous production, natural products, gene essentiality, minimized genome

## Abstract

*Streptomyces albus* J1074 is recognized as an effective host for heterologous production of natural products. Its fast growth and efficient genetic toolbox due to a naturally minimized genome have contributed towards its advantage in expressing biosynthetic pathways for a diverse repertoire of products such as antibiotics and flavonoids. In order to develop precise model-driven engineering strategies for de novo production of natural products, a genome-scale metabolic model (GEM) was reconstructed for the microorganism based on protein homology to model species *Streptomyces* *coelicolor* while drawing annotated data from databases and literature for further curation. To demonstrate its capabilities, the *Salb*-GEM was used to predict overexpression targets for desirable compounds using flux scanning with enforced objective function (FSEOF). *Salb*-GEM was also utilized to investigate the effect of a minimized genome on metabolic gene essentialities in comparison to another *Streptomyces* species, *S. coelicolor*.

## 1. Introduction

The use of actinomycete microbial cell factories has been paramount to the industrialization of bioactive compounds. Possessing all the essential metabolic components and biosynthetic machinery required for their production and proper modification (e.g., post-translational modification by CYP450 enzymes or phosphopantetheinyl transferases needed for proper function of polyketide synthases and non-ribosomal peptide synthetases), actinomycetes produce among the most diverse array of compounds [1,2,3]. Within this group of microorganisms, *Streptomyces albus* J1074 is commonly utilized as a microbial host for the heterologous production of bioactive natural products and secondary metabolites [4,5,6]. Two main factors culminating in preferential use of *S. albus* in biotechnological applications are its fast growth and an efficient genetic toolbox, which have proven it advantageous as a platform for product discovery and heterologous production [7]. *S. albus* J1074 was initially isolated as a derivative of the *S. albus* strain G defective in the *Sal*I restriction-modification system [8], which enabled the relatively straightforward genetic manipulation of the microorganism. Moreover, this streptomycete has relatively dispersed mycelial growth contrasting with the more common dense mycelial aggregation found in other species, which allows for better scaling up in an industrial setting.

There have been numerous endeavors to engineer *S. albus* J1074 (hereafter referred to solely as *S. albus*) to facilitate production of valuable compounds. The strain has been used for the heterologous expression of secondary biosynthetic gene clusters (BGC) taken from both *Streptomyces* and non-genus species such as the thiocoraline BGC from marine *Micromonospora* [9]. *S. albus* has been shown to harbor 22 BGCs, but clusters mostly remain cryptic (i.e., not expressed) across all stages of its growth in conventional liquid media [7,10,11,12]. Of the compounds known to be produced by *S. albus*, paulomycins are the only identified antibacterial compounds and their production remains highly unstable [13,14,15,16]. The known antifungal compounds produced endogenously consist of candicidins, antimycins, alteramides and surugamides [17]. Recently, it was also revealed that certain media such as SG2 was able to enhance production of these secondary metabolites [18], which was attributable to the overall increased transcriptional activity evidenced by RNA-seq studies on the organism grown in both SG2 and the more conventional TSB media. Additionally, novel strains of *S. albus* have been constructed with their native biosynthetic gene clusters removed [10], consequently leading not only to better compound detection (due to no background from endogenous secondary metabolites) but also to overall higher production yields compared to the ancestor *S. albus* strain and other *S. coelicolor* hosts. *S. albus* has been used to heterologously produce natural products including various types of flavonoids [19], and *S. albus* has been demonstrated to produce the flavonols myricetin, kaempferol and quercetin more efficiently than *S. coelicolor* [20].

However, despite the numerous studies on *S. albus,* there persists a lack of knowledge on how to best engineer the microbial host through a holistic approach. In order to glean a systems-level understanding of the metabolic potential of *S. albus*, a mathematical model of the organism metabolism can be developed and utilized. A genome-scale metabolic model (GEM) is a network-wide representation of the metabolism of the cell or microorganism [21,22]. GEMs serve as invaluable databases constructed from genome annotation, either inferentially or through experimentally validated gene-protein-reaction (GPR) relationships. This network connecting reactions and metabolites is mathematically delineated within a stoichiometric matrix. With the matrix as the main component, GEMs can be used as mathematical models to compute steady-state flux distributions and therefore predict phenotypes of the organism under different conditions. Furthermore, the GEM can be used as a canvas of sorts to contextualize “omics” data (e.g., proteomics, transcriptomics, and metabolomics) to derive further meaningful insights.

In order to further drive the engineering of *S. albus* as a chassis for heterologous compound production, understanding its capabilities and metabolism at the systems-wide level will yield insights by both integrating the current knowledge of the organism and informing new design strategies. To this end, the first GEM for *S. albus* J1074—herein denoted as *Salb*-GEM—was developed using *Sco*-GEM [23], a high-quality manually curated GEM for *S. coelicolor* (the most genetically well understood representative of the genus) as a template. In order to establish the use of this metabolic network reconstruction in metabolic engineering, this study assesses its capabilities relative to its reference model developed for *S. coelicolor* and demonstrates its applications in predicting gene targets for overproduction of target compounds. 

## 2. Results and Discussion

### 2.1. Reconstruction of Draft Genome-Scale Model

**Figure 1:**
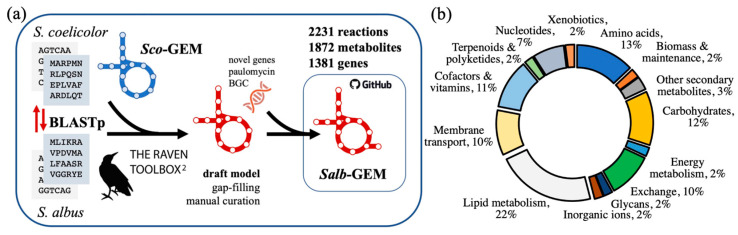
(**a**) overview of reconstruction and developmental pipeline of *Salb*-GEM. An initial draft model is developed using the RAVEN Toolbox with a protein FASTA of the *Streptomyces albus* J1074 genome and *Sco*-GEM as a template model. After manual curation and gap-filling of the metabolic network, additional genes and reactions specific to *S. albus* were incorporated externally from KEGG based on trained hidden Markov models (HMMs). *Salb*-GEM is distributed openly on GitHub, a version control and software development platform. (**b**) distribution of reactions sorted by metabolic subsystems.

*Salb*-GEM is based on the genome sequence of *S. albus* strain J1074. The reconstruction steps (Figure 1) leading up to the release of this model were carried out using the RAVEN toolbox [24,25], a MATLAB suite developed predominantly for genome-scale model reconstruction and constraint-based analysis. 

To first reconstruct the components of metabolism conserved among actinomycetes [26] or more specifically within the genus *Streptomyces*, the high-quality consensus GEM [23] of *S. coelicolor*, *Sco*-GEM, was referenced as a template model. Based on protein homology evaluated by bi-directional protein blast (BLASTp) between *S. albus* and *S. coelicolor* [27], a set of reactions associated with the identified orthologs was incorporated from *Sco*-GEM to form the initial draft network reconstruction of *Salb-*GEM. At this stage, 1645 metabolic reactions associated with 1278 genes were included into the initial model.

Subsequently, transport and spontaneous reactions with no specified gene associations were transferred from *Sco-*GEM to the draft model. Among these were exchange reactions and pseudo-reactions that contribute towards the representation of biomass within the model. Any reactions from this particular step that were disconnected from the remainder of the metabolic network were subsequently checked for and removed.

The resulting model remained unable to support growth via production of biomass. Consequently, gap-filling was implemented using the RAVEN toolbox, where a minimal number of reactions was transferred from the template model to enable the production of biomass components. From this step, an additional 18 genes and 119 reactions were incorporated into the model. A significant portion of these reactions did not have any explicit gene associations in the template model and were, therefore, not considered in the earlier semi-automated reconstruction steps. For example, mycothiol reductase is an essential reaction in thiol redox metabolism, which in *Streptomyces* involves the unique thiol mycothiol, and would therefore logically be part of the metabolic network reconstruction. However, the gene responsible for the enzymatic activity has not yet been experimentally verified and therefore remains in the model without gene association. A vast majority of these non-gene associated reactions that were added to the model were involved in lipid biosynthesis and fatty acid metabolism with multiple reactions corresponding to different levels of fatty acid chain saturation. In addition, the gap-filling identified reactions that were not captured in the first reconstruction steps, such as ATP synthase, as the stringent BLASTp parameters prevented matching of any subunits that were below the minimum length assigned. The resulting draft model was thereby able to produce all required biomass precursors.

### 2.2. Further Curation towards Salb-GEM

Following generation of the draft model, reactions and pathways specific to *S. albus* were incorporated in the model, thereby differentiating it from existing *Streptomyces* models. While there is little detailed and extensive knowledge of *S. albus* specific pathways available in literature, we applied an approach where hidden Markov models (HMMs) trained on the KEGG database [24] were used to query all *S. albus* proteins, and reactions associated with genes that were not already included in the draft model were thereby introduced. Through this process, 104 genes associated with 107 reactions were added to the metabolic network reconstruction. Although it is imperative for the GEM to provide a description of metabolism that is as comprehensive as possible, the majority of secondary metabolites and their associated pathways in *S. albus* remain relatively uncharacterized. While sequencing of the genome has revealed 22 BGCs, many of these clusters remain cryptic, typically silenced in conventional laboratory growth conditions [4,5,7]. As a result, the pathways associated with these clusters have yet to be elucidated for the various biochemical steps and precursors involved in product synthesis of a given cluster. 

Nonetheless, there have been experimental studies on the paulomycin BGC [13,28] that were able to provide detailed evidence towards its incorporation into the metabolic network reconstruction. *S. albus* produces the glycosylated antibiotics paulomycin A, B and E from activity of the paulomycin BGC. The pathway has been investigated by inactivation of genes encoding various enzymes, which are mainly involved in either transfer of glycosyl/acyl moieties or the biosynthesis of paulic acid and paulomycose. The study by Gonzalez et al. [14] has enabled the assignment of the genes to the individual steps along the biosynthetic pathway based on identification of products and intermediates accumulated in mutant strains defective in the aforementioned enzymatic steps. With this information on the proposed pathway steps and annotations also derived from KEGG, the corresponding reactions of the paulomycin pathway were manually introduced into the metabolic network reconstruction.

These various reconstruction steps culminated in the genome-scale model *Salb*-GEM comprising 2231 metabolic reactions, 1872 metabolites and 1381 genes (Table S1). The model is distributed through and maintained on a dedicated repository on GitHub (https://www.github.com/SysBioChalmers/Salb-GEM (accessed on 21 April 2021)), enabling open and reproducible curation and development, and allowing the issue of updated releases.

Within *Salb*-GEM, 126 reactions, 112 metabolites and 120 genes were exclusive to *S. albus* (i.e., not based on protein homology to *S. coelicolor*) as they were drawn from KEGG and manual curation of secondary metabolite pathways (e.g., paulomycin), indicating an anticipated significant overlap of metabolic pathways but also species-specific functionality. 

When referencing the pan-genome established for 17 *Streptomyces* species [29], 561 of the 1381 genes (40.6%) in *Salb*-GEM were part of the *Streptomyces* accessory genome. While the core genome denotes the portion of genes conserved among the 17 *Streptomyces* species, the accessory genome consists of genes whose presence differs considerably among *Streptomyces* species and confers upon them various advantageous traits to their environmental niches, generally providing functions that were not necessarily essential for viability. These 561 genes are associated to 729 reactions, with a majority of these reactions involved in secondary metabolism. With *Salb-*GEM containing a large fraction of accessory genes, it comprises substantially more than merely the conserved core metabolic pathways of *Streptomyces* but also acknowledges the diversity across species [30].

In addition to the inclusion of metabolic reactions, some of the macromolecule components (e.g., the protein, DNA and RNA pseudo-reactions) of the biomass reactions were successfully tailored based on the available genome and proteome of *S. albus*, while cellular DNA and protein content was measured as 0.035 and 21.95 (g/gDW), respectively, from an *S. albus* cultivation described below. However, to date no information is available on the contribution of other components to *S. albus* biomass including the composition of carbohydrate, lipid and any miscellaneous components that would need to be measured in relation to the total mass of the cell (as % of cell dry weight) before inclusion in the biomass reactions. Consequently, these macromolecular components of the biomass were assumed to be identical with *S. coelicolor* and therefore inferred from the *Sco*-GEM model. 

### 2.3. Calibrating Energetic Parameters from Cultivations

**Figure 2:**
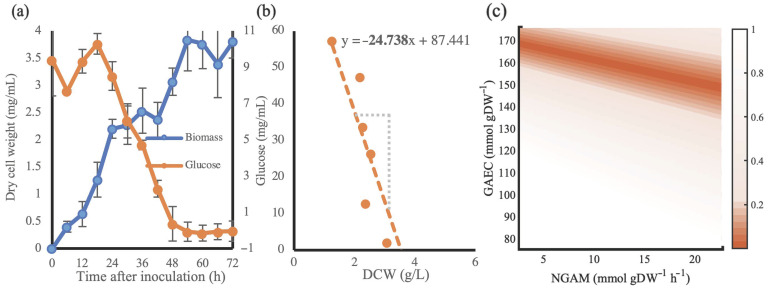
Cultivation data of *S. albus* J1074 grown on NL333 medium. (**a**) concentrations of dry cell weight of biomass (blue) and glucose (orange) in media sampled every 6 h over 72 h. (**b**) a plot of the recorded glucose levels (in mmol/L) and the corresponding biomass (in g/L) provides an estimate of the biomass yield. (**c**) comparison of deviations of predicted growth rates relative to the expected value of 0.04 h^−1^ across a combination of tested NGAM and GAEC values. Deviations within 100% of the expected value are shown in color.

To equilibrate the energy requirements of *S. albus* [31], a time-course cultivation of *S. albus* strain J1074 was performed, where glucose and biomass concentrations were monitored over 72 h (Figure 2a). The measurements from *t* = 18 h until *t* = 48 h were selected due to the clear trend of glucose consumption by an increasing cell mass. From combining these measurements transpired a biomass yield of 24.7 mmol glucose per 1 g dry cell weight (Figure 2b). Consequently, in *Salb*-GEM a glucose influx of 1 mmol/gDWh should result in a growth rate of 0.04 h^−1^. To equilibrate the model, the growth associated energy cost (GAEC) and non-growth associated maintenance (NGAM) can be fitted. While the exact distribution of energy costs across GAEC and NGAM could not be determined with the currently available experimental data, we were able to determine which combination of these energetic parameters result in the observed growth rate (Figure 2c). An NGAM of 6.86 mmol/gDCW/h and corresponding GAEC of 173 mmol/gDCW were selected to incorporate in the model.

### 2.4. Functional Comparison to Sco-GEM Reference Model

While *S. albus* and *S. coelicolor* are closely related, there are also profound differences in their genome and metabolism. *S. albus* has a smaller genome in comparison with *S. coelicolor*, containing 5832 and 7769 coding sequences respectively. Indeed, *S. albus* has among one of the smallest *Streptomyces* genomes at 6.84 Mbp while the size of the genome of *S. coelicolor* is 8.67 Mbp. A closer look into the genomic sequence also revealed that *S. albus* has a reduced number of orthologous group of genes. Furthermore, the single chromosome-based genome of *S. albus* encodes for seven rRNA operons contrary to the typical six associated with most *Streptomyces* species [7,32], which could possibly expound its fast growth rate. Such features have contributed to its use as a platform for heterologous production.

However, *Salb*-GEM has a slightly higher genomic coverage at 23.6% (with 1381 genes in the model) relative to the latter at a coverage of 22.9% (with 1777 genes), indicating the genome reduction in *S. albus* is not differentially affecting the number of metabolic genes. With regards to pathways that have been annotated in the model, *Salb*-GEM has demonstrably less reactions related to carbohydrate metabolism and secondary metabolic pathways but more so in relation to lipid metabolism.

Because *S. albus* has been remarked for its tendency to reduce the number of gene paralogues [7], there may as a result be a possible impact on the number of genes that are essential for viability, as lacking isoenzymes to catalyze reactions that are essential for viability would result in more essential genes. Therefore, we performed gene essentiality analysis with both *Salb*-GEM and *Sco*-GEM, and this revealed that *Salb*-GEM only has marginally more essential genes (185) than *Sco*-GEM (180) when set to grow on glucose minimal media, seemingly contradicting the reduced paralogues postulation. While a majority of the essential genes were associated with a common set of reactions in both organisms, certain genes were predicted to be uniquely essential for each organism, and their associated reactions were in distinct pathways (Tables S2 and S3).

More precisely, and in accordance with the reduced paralogues postulation, there were 77 reactions (Table S2) in *Salb*-GEM that have associations with an essential gene (which may be part of a complex but do not possess paralogues or isozymes) while the corresponding reaction relates to at least one paralogue in *Sco*-GEM. For instance, *Salb*-GEM possesses no paralogues for the 3-oxoacyl-(acyl carrier protein) synthase (XNR_4509), an important component responsible for the chain elongation step in fatty acid biosynthesis, but the same reaction in *Sco*-GEM is associated to four isozymes (SCO0548, SCO1266, SCO2390 and SCO3248). This gene alone is associated with 41 distinct metabolic reactions as each reaction corresponds to various degrees of saturation on the acyl moiety. Another example of differences in absence of paralogues is observed towards the end of the shikimate pathway, where the metabolic reactions mediated by 5-enolpyruvylshikimate-3-phosphate synthase, chorismate mutase, prephenate dehydrogenase and anthranilate phosphoribosyltransferase were associated with single genes in *Salb-*GEM unlike in *Sco*-GEM. Conversely, a total of 11 essential genes in *Sco-*GEM (associated to 29 reactions; Table S3) had paralogues to their *Salb*-GEM genes. The gene SCO6467 encoding for phosphatidylserine synthase in glycerophospholipid biosynthesis is essential in *Sco*-GEM, but its corresponding metabolic reaction in *Salb*-GEM is associated to two genes. Together, while the incidence of essential *Salb-*GEM genes with paralogues in *Sco*-GEM was twice higher than vice versa, sufficient cases could be found where the minimized genome of *Salb-*GEM still yielded more paralogues. This indicates that the reduced paralogue postulation is not necessarily true on a per-gene basis.

Beyond focusing on essential genes, it is feasible that the reduction in paralogues is not only associated with essential but also non-essential reactions and processes. For instance, nitrate reductase NarG is associated with only one gene cluster in *S. albus* while there are three known operons in *S. coelicolor* [7], while the activity of the *narG* genes is predicted to not be vital for growth. When systematically evaluating all gene-associated reactions in the GEMs of both species, 34.6% and 37.4% of those had gene associations involving paralogues in respectively *Salb*-GEM and *Sco*-GEM. The smaller genome in *S. albus* has indeed resulted in a somewhat reduced redundancy in isoenzymes that can catalyze the same reaction, but this reduction seemingly affects both essential and non-essential genes equally. Additionally, it is also noteworthy that this minimization of genetic duplicates may be more predominant in the context of regulation. The *S. albus* genome was reported to code for 35 sigma factors, which is smaller relative to the 65 sigma factors employed by *S. coelicolor.* The majority of these are of extra-cytoplasmic function, being responsible for responses to external stimuli such as stresses and used for morphology such as aerial mycelium development [7]. 

It is important to note that the biomass representation in *Salb*-GEM is partially derived from the *S. coelicolor* model due to the lack of available information on *S. albus* biomass composition. Any future curation of the biomass representation in *Salb*-GEM would have the potential to affect the results of gene essentiality analysis, however, it is anticipated that the complete description of *S. albus* biomass would not deviate drastically from that of *S. coelicolor*. Moreover, while certain macromolecular components such as the cell wall precursors may not have been experimentally determined for the organism, *S. albus*-specific gene associations were found for the synthesis of glycerol teichoic acid (contributing towards the formation of the bacterial cell wall) through manual curation [7].

### 2.5. Design Strategies for Improved Compound Production in S. albus

As *S. albus* is used for production of endogenous and heterologous compounds, it would be imperative for *Salb*-GEM to be able be used to design genetic strategies to improve productivity. Therefore, we applied flux scanning with enforced objective function (FSEOF) [33], an algorithm that identifies reactions as targets for modulation of gene expression based on the relationship of flux through the reaction and an incrementally increased flux towards compound production. Typically, genes associated with reactions that positively correlate to compound production are identified as potential targets for overexpression. The strength of this correlation, reflected by the *slope* in the FSEOF analysis (Table S4), is indicative of how beneficial the overexpression will be for improved compound production.

To query a range of potential products, we selected three compounds related to the endogenous BGCs in *S. albus* for FSEOF analysis, namely paulomycin, antimycin and candicidin, in addition to two heterologous natural products, namely the thiodepsipeptide thiocoraline [9] originating from *Micronospora* actinomycetes and naringenin, a flavanone from which can be derived a majority of flavonoid compounds [34].

When considering the various genetic targets that were predicted by FSEOF to increase productivity, subsets of various common genes were predicted for different combinations of the five endogenous and heterologous compounds. Ten genetic targets were identified as positively affecting all product compounds, of which seven targets covered the majority of the shikimate pathway (i.e., biosynthesis of aromatic amino acids L-phenylalanine, L-tyrosine and L-tryptophan), and the remaining three targets were associated with the pentose phosphate pathway. It is worth to note that the three reactions of the pentose phosphate pathway are responsible for the availability of erythrose 4-phosphate, which along with phosphoenolpyruvate from the glycolytic pathway is the direct precursor to the first metabolic reaction step of the shikimate pathway via the 3-deoxy-D-arabino-heptulosonate 7-phosphate synthetase.

**Figure 3:**
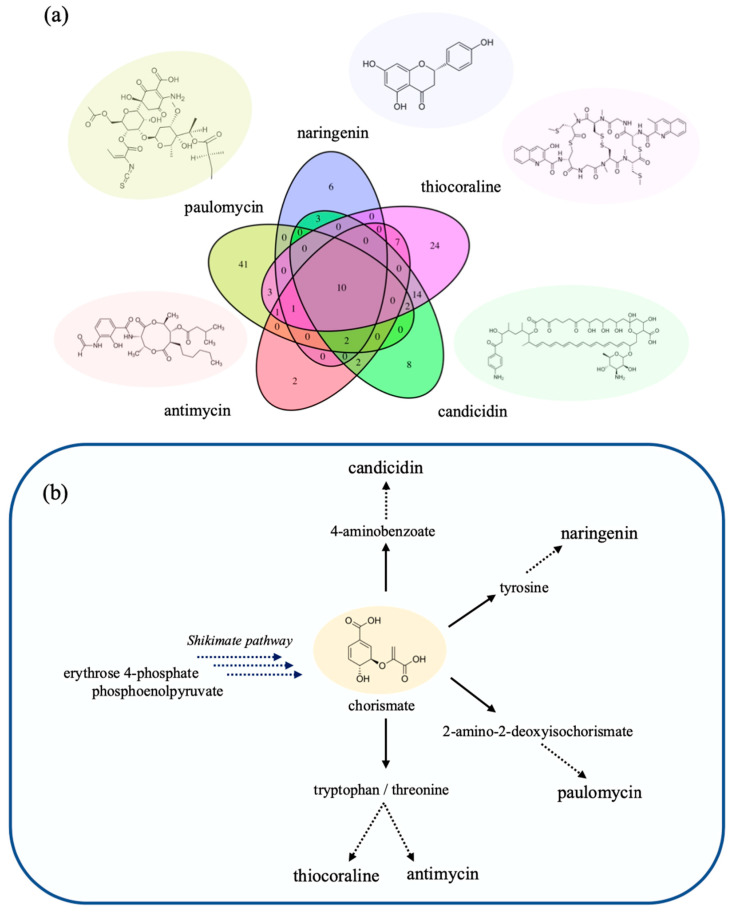
Overview of FSEOF to predict engineering targets for improved product synthesis. (**a**) a comparison of listed targets (reactions) predicted for each product and between how many targets the suggested targets are shared. Further details on reaction name, pathway and *slope* can be found in Table S3. (**b**) simplified view of pathways branching off from the shikimate pathway towards individual product targets. Each product draws from chorismate as a precursor.

Considering the structure of each target compound (Figure 3a), which contains a myriad of aromatic moieties, it can be anticipated that all target compounds benefit from an increased expression of the shikimate pathway, as each of the targets counts chorismate among its precursors. For example, the biosynthesis of the antibacterial paulomycin converts chorismate to 2-amino-2-deoxyisochorismate as the first step in the pathway. In the cases of thiocoraline and the antifungal antimycin, the chorismate branches off towards synthesis of threonine and tryptophan. For the synthesis of the antifungal candicidin, the chorismate is converted into the 4-aminobenzoate, which is also a precursor to folate. Lastly, the heterologous production of naringenin utilizes tyrosine or phenylalanine depending on which exogenous enzyme is used. This can be seen where the enzymes chorismate mutase, prephenate dehydrogenase and tyrosine transaminase are uniquely predicted for the improved production of naringenin (Figure 3b).

In addition, significant overlap across multiple compounds could be observed as genetic targets that function in fatty acid biosynthesis. The production of paulomycin, naringenin and candicidin which draw fatty acyl-CoA precursors (e.g., crotonyl-CoA and malonyl-CoA) from primary metabolism, would benefit from the overexpression of fatty acid biosynthetic genes. This can likely be further extrapolated to all polyketide secondary metabolites that draw heavily from these precursors. In the case of naringenin and candicidin, which both utilize malonyl-CoA as precursor, among the overexpression targets carrying the most flux was acetyl-CoA carboxylase, a reaction that converts acetyl-CoA to malonyl-CoA by condensation [35,36]. Indeed, in the context of naringenin, malonyl-CoA been cited as the predominant limiting factor in further production of flavonoid compounds [19,20,37]. 

While the targets share many common reactions in the shikimate pathway and fatty acid biosynthesis, there is also a myriad of unique targets that differ starkly in regard to their associated pathways such as the previously mentioned overexpression of prephenate dehydrogenase (XNR_5061) to convert chorismate to L-tyrosine, a precursor leading to naringenin. With regards to another instance, in order to augment the availability of the L-paulomycose moiety on the basic paulomycin structure, many suggested targets originate from purine and pyrimidine metabolism. 

For other compounds such as thiocoraline that draw heavily from L-cysteine as a building block, suggested targets also encompass cysteine and sulfur metabolism such as sulfate adenyltransferase (XNR_0714 and XNR_0715) and adenylyl-sulfate kinase (XNR_0713). Other compounds that are produced from endogenous BGCs such as candicidin also reflect the role that fatty acid precursors have in limiting overall yields. For instance, in order to improve precursor flux towards candicidin biosynthesis, the *(R)*-Methylmalonyl-CoA CoA-carbonylmutase (XNR_4666) and methylmalonyl-CoA epimerase (XNR_1439) are beneficial enzymes to overexpress.

Of the various predicted overexpression targets, few have been reportedly tested to increase production formation in *S. albus.* Interestingly, the overexpression of acetyl-CoA carboxylase, beneficial for both naringenin and candicidin production according *Salb*-GEM, has been investigated in *S. albus* for increased production of the flavonol myricetin, which has naringenin as a precursor. Subunits of the acetyl-CoA carboxylase complex from *S. coelicolor* were heterologously expressed in *S. albus* [20]. As the complex converts acetyl-CoA to malonyl-CoA, higher productivity of myricetin was anticipated. Indeed, flavonol production increased, but not in the form of myricetin but rather in the detection of the shunt product apigenin. This highlights that the FSEOF approach is solely based on the metabolic network stoichiometry and does not factor in modes of regulation. Meanwhile, overexpression of the tyrA and aroG genes in *Escherichia coli*, coding for chorismate synthase/prephenate dehydrogenase (CHORM and PPND) and 3-deoxy-D-arabino-heptulosonate 7-phosphate synthetase (DDPA), yielded increased naringenin production [38]. While FSEOF applied to *Salb*-GEM indeed provides effective strategies to facilitate precursor flux from primary metabolism, an important limitation remains the limited detailed information that is available of the heterologous pathway themselves, precluding inclusion in the model.

## 3. Materials and Methods

### 3.1. Model Reconstruction

#### 3.1.1. Homology-Based Reconstruction

The genome-scale metabolic network reconstruction for *S. albus* J1074 was based on published genome annotation (GenBank: CP004370.1, NCBI: PRJNA180996) and carried out using the RAVEN Toolbox [24,25]. By referencing the most up-to-date and complete GEM of *S. coelicolor*, *Sco*-GEM [23], as a template model, the first draft of the GEM was created by assigning reactions based on protein homology. This was performed by running the RAVEN function *getModelFromHomology*, which utilizes the data structure from a bi-directional homology search via BLASTp and assigns the best-fitting GPRs using the following parameters as cut-offs: a maximum E-value of 10^−50^, a minimum alignment length of 90 residues and a minimum identity of 40%. These values for the parameters have been selected in order to incorporate the reactions essential for various growth phenotypes and minimize the number of reactions added by the gap-filling process in later steps. Any spontaneous reactions or transport reactions with no gene-association were also imported from the *Sco*-GEM template model.

#### 3.1.2. Definition of Biomass Composition and Energetic Parameters

Where no experimental data was available for biomass components in *S. albus*, it was assumed that these would not deviate drastically from *S. coelicolor*. Therefore, the storage carbohydrate, cell wall and lipid components of the biomass reaction were taken from *Sco*-GEM, while the stoichiometric coefficients of ribonucleotides, nucleotides and amino acids were inferred from the *S. albus* genome and coding sequences. The DNA and protein content were determined from cultivations reported in this work, while also the energetic parameters GAEC and NGAM were taken from these. While the experimental data only allowed for determining the sum of GAEC and NGAM, the NGAM was set at 6.86 mmol/gDCW/h in agreement with the model prokaryotic GEM of *Escherichia coli*, resulting with a GAEC of 173 mmol/gDCW.

#### 3.1.3. Gap-Filling

To yield a functional model, gap-filling was performed using RAVEN function *fillGaps* to retrieve the minimum set of reactions from *Sco*-GEM to ensure that the model can grow in silico. After the initial gap-filling, the model was evaluated for growth solely on other carbon sources (e.g., fructose, xylose, and mannitol). For substrates that are known to support *S. albus* growth but the phenotype with which could not be replicated in silico, the minimum number of reactions were added from the template model to support utilization of the designated substrate. For latter substrates, manual gap-filling was performed by adding reactions that carried flux in *Sco*-GEM solely utilizing the corresponding substrates to the draft model; any new reactions that did not carry flux in the model were subsequently removed. Additional reactions were incorporated from another draft model generated from KEGG via RAVEN function *getKEGGModelForOrganism*, which utilizes HMMER in homology search and queries the *S. albus* proteome against KEGG orthology (KO)-trained hidden Markov models (HMMs). The additional reactions were checked to not be associated with any genes already included in the previous draft model.

#### 3.1.4. Curation of Reaction Reversibility

Reactions added from this and later steps were subsequently curated for directionality. eQuilibrator [39] was used to determine reversibility for the additional reactions based on computed Gibbs free energy values at standard conditions (25 °C, 1 bar), pH7 and 1mM concentration of reactants. A threshold of −30 kJ/mol was applied to define a reaction as irreversible. For any cases wherein the Gibbs free energy could not be calculated (e.g., primarily from metabolites not recognized by the API), the reactions were curated manually by checking that they do not contribute to any futile cycles.

#### 3.1.5. Model Distribution

The development and curation of *Salb*-GEM is reproducibly tracked and freely available from https://www.github.com/SysBioChalmers/Salb-GEM (accessed on 21 April 2021), and archived on Zenodo (https://doi.org/10.5281/zenodo.3693405 (accessed on 21 April 2021)). The model is versioned, and release 1.0.1 was used in the analyses presented here. The model is distributed in SBML L3V1 FBCv2 format, while an overview of the reactions in version 1.0.1 is also shown in Table S1.

### 3.2. Model Analysis

Within the feasible solution space at steady-state, it is possible to further identify specific points (e.g., flux distributions) that satisfy certain conditions specified as objective functions to be either maximized or minimized. In most cases, this objective function is based on the biomass reaction representing the organism’s main goal to grow as fast as possible. A commonly used mathematical method to analyze metabolic network reconstructions is flux balance analysis (FBA)**,** which calculates the flow, or flux, of metabolites through the metabolic network, enabling prediction of an organism’s growth rate or production rate of a desired metabolite.

Gene essentiality analysis was carried out using the *singleGeneDeletion* function on COBRA Toolbox [40]. Essential genes were determined by comparing the in silico growth rate of the gene knockout mutant with that of the wild-type strain. Any gene of which the knockout consequently leds to a reduction in the growth prediction by two-thirds or more if not leading to no growth entirely (e.g., an infeasible problem) is categorized as essential.

Potential targets for metabolic engineering towards improved yield of natural products were predicted by using flux scanning with enforced objective function (FSEOF) [33]. The method works by scanning all metabolic fluxes in the GEM and prioritizing fluxes that increase when the flux towards product is enforced as a constraint during flux balance analysis while maximizing biomass formation flux as well.

### 3.3. Cultivation and Data Collection

Triplicate cultures of *S. albus* J1074 were grown in shake flasks with 100 mL of NL333 medium [5] over a duration of 72 h, using an inoculum of 10^8^ CFU/mL. Samples were taken every 6 h for glucose concentrations as determined using glucose oxidase colorometric assay (GAGO-20, Sigma-Aldrich, Gillingham, UK) following manufacturer’s instructions; biomass determined as dry cell weight, protein content by Bradford assay as previously described [19] and DNA concentration by Burton method [41].

## 4. Conclusions

Here we present the first genome-scale metabolic model (GEM) for *Streptomyces albus* J1074, which can be used in constraint-based analysis. We demonstrated utility of the model in identifying sets of shared targets that may be tested to improve endogenous and heterologous production in *S. albus*. Additionally, we have used the model to glean insights into the effect of the *S. albus* minimal genome on its metabolism, comparing the existence of paralogues between *S. albus* and *S. coelicolor* and the resulting impact on gene essentiality. The *Salb*-GEM model was able to provide realistic growth predictions in comparison with experimentally measured growth and glucose consumption rates. However, the model would benefit from a more detailed description of the *S. albus* biomass composition, evidence of gene essentiality and general fermentation data. When such relevant experimental data will become available, the model can readily be curated and further improved. Open and reproducible model curation and distribution of an updated model are facilitated by continually hosting the model on GitHub (https://www.github.com/SysBioChalmers/Salb-GEM (accessed on 21 April 2021)), where the research community can transparently reference, reproduce and contribute to the model curation and development.

## Data Availability

The model files, reconstruction pipeline and relevant data can be found at http://www.github.com/SysBioChalmers/Salb-GEM (accessed on 21 April 2021).

## Supplementary Materials

The following are available online at https://www.mdpi.com/article/10.3390/metabo11050304/s1, Table S1: Metabolic reactions and their gene association as represented in Salb-GEM. Table S2: Metabolic reactions in *Salb*-GEM and *Sco*-GEM associated with essential genes predicted in Salb-GEM with multiple paralogues in *Sco*-GEM, Table S3: Metabolic reactions in *Salb*-GEM and *Sco*-GEM associated with essential genes predicted in *Sco*-GEM with multiple paralogues in *Salb*-GEM. Table S4: Summary of predicted FSEOF targets sorted by product of interest.
