# Supplementary material for: Reconstruction of a Genome-Scale Metabolic Model of Streptomyces albus J1074: Improved Engineering Strategies in Natural Product Synthesis
Source: Metabolites. 2021 May 11;11(5):304. doi: 10.3390/metabo11050304 (PMC8150979; doi:10.3390/metabo11050304)
Supplement: Supplementary file 1 [file metabolites-11-00304-s001.zip › TableS4_new.pdf]

Target product	Slope	Reaction ID	Reaction Name	Pathway	GPR
naringenin	2.80	FNOR	putative ferredoxin/ferredoxin-NADP reductase	FERI metabolism	XNR_2490
naringenin	2.76	ACCOAC	acetyl-CoA carboxylase	Fatty acid biosynthesis	(XNR_2648 and XNR_4211) or (XNR_2648 and XNR_2273) or (XNR_2648 and XNR_4019) or (XNR_1278 and XNR_1277 and XNR_4211) or (XNR_1278 and XNR_1277 and XNR_2273) or (XNR_1278 and XNR_1277 and XNR_4019)
naringenin	2.68	ENO	enolase	Glycolysis/Gluconeogenesis	XNR_2005 or XNR_2219
naringenin	2.68	PGM	phosphoglycerate mutase	Glycolysis/Gluconeogenesis	XNR_3385 or XNR_4361
naringenin	2.60	PYRS	pyruvate synthase	Pyruvate metabolism	XNR_3672 or XNR_3673
naringenin	2.58	HCO3E	HCO3 equilibration reaction	Nitrogen metabolism	XNR_2509 or XNR_2719 or XNR_4786
naringenin	1.16	PYK	pyruvate kinase	Glycolysis/Gluconeogenesis	XNR_1410 or XNR_4867
naringenin	0.98	PPND	prephenate dehydrogenase	Phenylalanine, tyrosine and tryptophan biosynthesis	XNR_5061
naringenin	0.98	TYRTA	tyrosine transaminase	Phenylalanine, tyrosine and tryptophan biosynthesis	XNR_2937 or XNR_3189 or XNR_3703 or XNR_4825 or XNR_4967
naringenin	0.95	CHORM	chorismate mutase	Phenylalanine, tyrosine and tryptophan biosynthesis	XNR_4859
naringenin	0.92	CHORS	chorismate synthase	Phenylalanine, tyrosine and tryptophan biosynthesis	XNR_5355
naringenin	0.92	DDPA	3-deoxy-D-arabino-heptulosonate 7-phosphate synthetase	Phenylalanine, tyrosine and tryptophan biosynthesis	XNR_0595 or XNR_4763
naringenin	0.92	DHQS	3-dehydroquinate synthase	Phenylalanine, tyrosine and tryptophan biosynthesis	XNR_5357
naringenin	0.92	DHQTi	3-dehydroquinate dehydratase, irreversible	Phenylalanine, tyrosine and tryptophan biosynthesis	XNR_4909
naringenin	0.92	PSCVT	3-phosphoshikimate 1-carboxyvinyltransferase	Phenylalanine, tyrosine and tryptophan biosynthesis	XNR_1588
naringenin	0.92	SHK3Dr	shikimate dehydrogenase	Phenylalanine, tyrosine and tryptophan biosynthesis	XNR_5354
naringenin	0.92	SHKK	shikimate kinase	Phenylalanine, tyrosine and tryptophan biosynthesis	XNR_5356
naringenin	0.53	TKT2	transketolase	Pentose phosphate pathway	XNR_4929
naringenin	0.50	PPDK	Pyruvate,phosphate dikinase	Glycolysis/Gluconeogenesis	XNR_4449
naringenin	0.23	TPI	triose-phosphate isomerase	Glycolysis/Gluconeogenesis	XNR_4921

naringenin	0.14	RPE	ribulose 5-phosphate 3-epimerase	Pentose phosphate pathway	XNR_5386
naringenin	0.14	RPI	ribose-5-phosphate isomerase	Pentose phosphate pathway	XNR_4316
paulomycin	2.77	GALM1	Aldose 1-epimerase	Glycolysis/Gluconeogenesis	XNR_1676
paulomycin	2.77	GLUKB	glucokinase-bglc	Glycolysis/Gluconeogenesis	XNR_0083 or XNR_0553 or XNR_2406 or XNR_4754
paulomycin	2.06	NDPK2	nucleoside-diphosphate kinase (ATP:UDP)	Pyrimidine metabolism	XNR_4333
paulomycin	2.00	G1PTT	dTTP:alpha-D-glucose-1-phosphate thymidylyltransferase	Polyketide sugar unit biosynthesis	XNR_0593
paulomycin	1.98	FMNRx2	FMN reductase	Riboflavin metabolism	XNR_0710 or XNR_3551
paulomycin	1.98	NDPK4	nucleoside-diphosphate kinase (ATP:dTDP)	Pyrimidine metabolism	XNR_4333
paulomycin	1.60	GALUi	UTP-glucose-1-phosphate uridylyltransferase (irreversible)	Amino sugar and nucleotide sugar metabolism	XNR_2129
paulomycin	1.41	TRE6PS	alpha,alpha-trehalose-phosphate synthase (UDP-forming)	Starch and sucrose metabolism	XNR_2398
paulomycin	1.41	TRE6PPP	trehalose-6-phosphate phosphorylase	Starch and sucrose metabolism	XNR_1712
paulomycin	1.00	ACACT1r	acetyl-CoA C-acetyltransferase	Fatty acid biosynthesis	XNR_0217 or XNR_0301 or XNR_1438 or XNR_1987 or XNR_2158 or XNR_3582
paulomycin	1.00	DDALLO	dTDP-(2R,6S)-6-hydroxy-2-methyl-3-oxo-3,6-dihydro-2H-pyran-4-olate 3-ketoreductase	Polyketide sugar unit biosynthesis	XNR_0575 or XNR_2309
paulomycin	1.00	ECOAH1	3-hydroxyacyl-CoA dehydratase (3-hydroxybutanoyl-CoA)	Fatty acid biosynthesis	XNR_0300 or XNR_2271
paulomycin	1.00	HBCO_nadp	3-hydroxybutyryl-CoA dehydrogenase	Butanoate metabolism	XNR_0454 or XNR_1452 or XNR_5241
paulomycin	1.00	TDPGDH	dTDPglucose 4,6-dehydratase	Amino sugar and nucleotide sugar metabolism	XNR_0594
paulomycin	0.99	ADNK1	adenosine kinase	Purine metabolism	XNR_4722
paulomycin	0.99	AHCi	adenosylhomocysteinase	Cysteine and methionine metabolism	XNR_1908
paulomycin	0.98	DTMPK	dTMP kinase	Pyrimidine metabolism	XNR_2700
paulomycin	0.98	DUTPDP	dUTP diphosphatase	Pyrimidine metabolism	XNR_1004
paulomycin	0.98	NDPK6	nucleoside-diphosphate kinase (ATP:dUDP)	Pyrimidine metabolism	XNR_4333
paulomycin	0.98	TMDSf	thymidylate synthase (Flavin-dependent)	Pyrimidine metabolism	XNR_1124

paulomycin	0.95	METAT	methionine adenosyltransferase	Methionine metabolism	XNR_5375
paulomycin	0.91	METS	methionine synthase	Methionine metabolism	XNR_5171
paulomycin	0.90	MTHFR3_1	Methylenetetrahydrofolate reductase (NADPH)	Folate metabolism	XNR_4775
paulomycin	0.79	CHORS	chorismate synthase	Phenylalanine, tyrosine and tryptophan biosynthesis	XNR_5355
paulomycin	0.79	DDPA	3-deoxy-D-arabino-heptulosonate 7-phosphate synthetase	Phenylalanine, tyrosine and tryptophan biosynthesis	XNR_0595 or XNR_4763
paulomycin	0.79	DHQS	3-dehydroquinate synthase	Phenylalanine, tyrosine and tryptophan biosynthesis	XNR_5357
paulomycin	0.79	DHQTi	3-dehydroquinate dehydratase, irreversible	Phenylalanine, tyrosine and tryptophan biosynthesis	XNR_4909
paulomycin	0.79	PSCVT	3-phosphoshikimate 1-carboxyvinyltransferase	Phenylalanine, tyrosine and tryptophan biosynthesis	XNR_1588
paulomycin	0.79	SHK3Dr	shikimate dehydrogenase	Phenylalanine, tyrosine and tryptophan biosynthesis	XNR_5354
paulomycin	0.79	SHKK	shikimate kinase	Phenylalanine, tyrosine and tryptophan biosynthesis	XNR_5356
paulomycin	0.77	ASPCT	aspartate carbamoyltransferase	Pyrimidine metabolism	XNR_5364
paulomycin	0.77	DHORTS	dihydroorotase	Pyrimidine metabolism	XNR_5365
paulomycin	0.77	OMPDC	orotidine-5'-phosphate decarboxylase	Pyrimidine metabolism	XNR_5370
paulomycin	0.77	ORPT	orotate phosphoribosyltransferase	Pyrimidine metabolism	XNR_3194
paulomycin	0.76	PPDK	Pyruvate,phosphate dikinase	Glycolysis/Gluconeogenesis	XNR_4449
paulomycin	0.68	APAT2r	3-Aminopropanoate:2-oxoglutarate aminotransferase	beta-Alanine metabolism	XNR_1169 or XNR_2767
paulomycin	0.68	ASP1DC	aspartate 1-decarboxylase	beta-Alanine metabolism	XNR_0119 or XNR_3726 or XNR_4602
paulomycin	0.68	MMSAD3	methylmalonate-semialdehyde dehydrogenase (malonic semialdehyde)	Propanoate metabolism	XNR_0154 or XNR_4241
paulomycin	0.68	UMPK	UMP kinase	Pyrimidine metabolism	XNR_1198
paulomycin	0.65	TKT2	transketolase	Pentose phosphate pathway	XNR_4929
paulomycin	0.64	GLNS	glutamine synthetase	Alanine, aspartate and glutamate metabolism	XNR_4631 or XNR_4658 or XNR_4684 or XNR_5219
paulomycin	0.54	CBPS	carbamoyl-phosphate synthase (glutamine-hydrolysing)	Arginine and proline metabolism	XNR_5368 and XNR_5367

paulomycin	0.52	RPI	ribose-5-phosphate isomerase	Pentose phosphate pathway	XNR_4316
paulomycin	0.51	RPE	ribulose 5-phosphate 3-epimerase	Pentose phosphate pathway	XNR_5386
paulomycin	0.38	PRPPS	phosphoribosylpyrophosphate synthetase	Purine metabolism	XNR_2061
paulomycin	0.13	ENO	enolase	Glycolysis/Gluconeogenesis	XNR_2005 or XNR_2219
paulomycin	0.13	PGM	phosphoglycerate mutase	Glycolysis/Gluconeogenesis	XNR_3385 or XNR_4361
antimycin	0.98	ANPRT	anthranilate phosphoribosyltransferase	Phenylalanine, tyrosine and tryptophan biosynthesis	XNR_4733
antimycin	0.98	ATNS_nh4	chorismate pyruvate-lyase	Phenylalanine, tyrosine and tryptophan biosynthesis	XNR_3034 or (XNR_5862 and XNR_4836) or XNR_3034 or (XNR_5862 and XNR_4836)
antimycin	0.98	IGPS	indole-3-glycerol-phosphate synthase	Phenylalanine, tyrosine and tryptophan biosynthesis	XNR_4840
antimycin	0.98	PRAIi	phosphoribosylanthranilate isomerase (irreversible)	Phenylalanine, tyrosine and tryptophan biosynthesis	XNR_4829
antimycin	0.98	TRPS1	tryptophan synthase (indoleglycerol phosphate)	Tryptophan metabolism	XNR_4842 and XNR_4841
antimycin	0.92	HSK	homoserine kinase	Glycine, serine and threonine metabolism	XNR_1477
antimycin	0.92	THRS	threonine synthase	Glycine, serine and threonine metabolism	XNR_1478 or XNR_2396
antimycin	0.91	CHORS	chorismate synthase	Phenylalanine, tyrosine and tryptophan biosynthesis	XNR_5355
antimycin	0.91	DDPA	3-deoxy-D-arabino-heptulosonate 7-phosphate synthetase	Phenylalanine, tyrosine and tryptophan biosynthesis	XNR_0595 or XNR_4763
antimycin	0.91	DHQS	3-dehydroquinate synthase	Phenylalanine, tyrosine and tryptophan biosynthesis	XNR_5357
antimycin	0.91	DHQTi	3-dehydroquinate dehydratase, irreversible	Phenylalanine, tyrosine and tryptophan biosynthesis	XNR_4909
antimycin	0.91	PSCVT	3-phosphoshikimate 1-carboxyvinyltransferase	Phenylalanine, tyrosine and tryptophan biosynthesis	XNR_1588
antimycin	0.91	SHK3Dr	shikimate dehydrogenase	Phenylalanine, tyrosine and tryptophan biosynthesis	XNR_5354
antimycin	0.91	SHKK	shikimate kinase	Phenylalanine, tyrosine and tryptophan biosynthesis	XNR_5356
antimycin	0.84	TKT2	transketolase	Pentose phosphate pathway	XNR_4929
antimycin	0.81	ASAD	aspartate-semialdehyde dehydrogenase	Threonine and Lysine metabolism	XNR_2758 or XNR_4304
antimycin	0.81	ASPK	aspartate kinase	Alanine, aspartate and glutamate metabolism	XNR_2759

antimycin	0.79	RPI	ribose-5-phosphate isomerase	Pentose phosphate pathway	XNR_4316
antimycin	0.78	RPE	ribulose 5-phosphate 3-epimerase	Pentose phosphate pathway	XNR_5386
antimycin	0.72	PRPPS	phosphoribosylpyrophosphate synthetase	Purine metabolism	XNR_2061
antimycin	0.43	PPDK	Pyruvate phosphate dikinase	Glycolysis/Gluconeogenesis	XNR_4449
antimycin	0.36	ENO	enolase	Glycolysis/Gluconeogenesis	XNR_2005 or XNR_2219
antimycin	0.36	PGM	phosphoglycerate mutase	Glycolysis/Gluconeogenesis	XNR_3385 or XNR_4361
antimycin	0.20	PGI	glucose-6-phosphate isomerase	Glycolysis/Gluconeogenesis	XNR_4924
candicidin	13.90	ACCOAC	acetyl-CoA carboxylase	Fatty acid biosynthesis	(XNR_2648 and XNR_2273) or XNR_4211 or (XNR_2648 and XNR_2273) or XNR_4019 or XNR_4211 or (XNR_2648 and XNR_2273) or XNR_4019 or XNR_4211
candicidin	13.08	HCO3E	HCO3 equilibration reaction	Nitrogen metabolism	XNR_2509 or XNR_2719 or XNR_4786
candicidin	9.51	ENO	enolase	Glycolysis/Gluconeogenesis	XNR_2005 or XNR_2219
candicidin	9.51	PGM	phosphoglycerate mutase	Glycolysis/Gluconeogenesis	XNR_3385 or XNR_4361
candicidin	6.38	HEX1	glucokinase (D-glucose:ATP)	Starch and sucrose metabolism	XNR_0553 or XNR_4754 or XNR_0553 or XNR_4754 or XNR_0083 or (XNR_3293 and XNR_4146) or XNR_2406
candicidin	4.00	MME	methylmalonyl-CoA epimerase	Valine, leucine and isoleucine degradation	XNR_1439
candicidin	4.00	MMM2	(R)-Methylmalonyl-CoA CoA-carbonylmutase	Valine, leucine and isoleucine degradation;Glyoxylate and dicarboxylate metabolism;Propanoate metabolism;Carbon fixation pathways in prokaryotes	XNR_4666
candicidin	2.56	GAPD	glyceraldehyde-3-phosphate dehydrogenase	Glycolysis/Gluconeogenesis	XNR_0959 or XNR_3771 or XNR_4919
candicidin	2.56	PGK	phosphoglycerate kinase	Glycolysis/Gluconeogenesis	XNR_4920
candicidin	1.55	TPI	triose-phosphate isomerase	Glycolysis/Gluconeogenesis	XNR_4921
candicidin	1.11	FBA	fructose-bisphosphate aldolase	Glycolysis/Gluconeogenesis	XNR_2411 or XNR_3195
candicidin	1.11	PFK	phosphofructokinase	Glycolysis/Gluconeogenesis	XNR_1407 or XNR_5592
candicidin	1.00	MTHFC	methenyltetrahydrofolate cyclohydrolase	Folate metabolism	XNR_3872 or XNR_5257
candicidin	1.00	MTHFD	methylenetetrahydrofolate dehydrogenase (NADP)	Folate metabolism	XNR_3872 or XNR_5257
candicidin	0.97	ADCL	4-aminobenzoate synthase	Folate biosynthesis	XNR_5309
candicidin	0.97	ADCS	4-amino-4-deoxychorismate synthase	Folate biosynthesis	XNR_3034 or XNR_5308 or XNR_5862

candicidin	0.78	PGI	glucose-6-phosphate isomerase	Glycolysis/Gluconeogenesis	XNR_4924
candicidin	0.73	ADSL1r	adenylsuccinate lyase	Purine metabolism	XNR_5550
candicidin	0.73	ADSS	adenylosuccinate synthase	Purine metabolism	XNR_3210
candicidin	0.63	CHORS	chorismate synthase	Phenylalanine, tyrosine and tryptophan biosynthesis	XNR_5355
candicidin	0.63	DDPA	3-deoxy-D-arabino-heptulosonate 7-phosphate synthetase	Phenylalanine, tyrosine and tryptophan biosynthesis	XNR_0595 or XNR_4763
candicidin	0.63	DHQS	3-dehydroquinate synthase	Phenylalanine, tyrosine and tryptophan biosynthesis	XNR_5357
candicidin	0.63	DHQTi	3-dehydroquinate dehydratase, irreversible	Phenylalanine, tyrosine and tryptophan biosynthesis	XNR_4909
candicidin	0.63	PSCVT	3-phosphoshikimate 1-carboxyvinyltransferase	Phenylalanine, tyrosine and tryptophan biosynthesis	XNR_1588
candicidin	0.63	SHK3Dr	shikimate dehydrogenase	Phenylalanine, tyrosine and tryptophan biosynthesis	XNR_5354
candicidin	0.63	SHKK	shikimate kinase	Phenylalanine, tyrosine and tryptophan biosynthesis	XNR_5356
candicidin	0.57	GLNS	glutamine synthetase	Alanine, aspartate and glutamate metabolism	XNR_4631 or XNR_4658 or XNR_4684 or XNR_5219
candicidin	0.56	ADSL2r	adenylosuccinate lyase	Purine metabolism	XNR_5550
candicidin	0.56	AIRCr	phosphoribosylaminoimidazole carboxylase	Purine metabolism	XNR_1959 and XNR_1960
candicidin	0.56	GARFT	phosphoribosylglycinamide formyltransferase	Purine metabolism	XNR_3869
candicidin	0.56	GLUPRT	glutamine phosphoribosyldiphosphate amidotransferase	Purine metabolism	XNR_2842
candicidin	0.56	PRAGSr	phosphoribosylglycinamide synthase	Purine metabolism	XNR_2857
candicidin	0.56	PRAIS	phosphoribosylaminoimidazole synthase	Purine metabolism	XNR_2841
candicidin	0.56	PRASCSi	phosphoribosylaminoimidazolesuccinocarboxamide synthase	Purine metabolism	XNR_2854
candicidin	0.56	PRFGS	phosphoribosylformylglycinamidine synthase	Purine metabolism	XNR_2844 and XNR_2845 and XNR_2846
candicidin	0.44	AICART	phosphoribosylaminoimidazolecarboxamide formyltransferase	Purine metabolism	XNR_3870
candicidin	0.44	IMPC	IMP cyclohydrolase	Purine metabolism	XNR_3870

candicidin	0.38	TKT2	transketolase	Pentose phosphate pathway	XNR_4929
candicidin	0.15	RPI	ribose-5-phosphate isomerase	Pentose phosphate pathway	XNR_4316
candicidin	0.13	RPE	ribulose 5-phosphate 3-epimerase	Pentose phosphate pathway	XNR_5386
thiocoraline	38.73	ASPT	aspartate ammonia-lyase	Alanine, aspartate and glutamate metabolism	XNR_4132
thiocoraline	17.71	MTHFC	methenyltetrahydrofolate cyclohydrolase	Folate metabolism	XNR_3872 or XNR_5257
thiocoraline	17.71	MTHFD	methylenetetrahydrofolate dehydrogenase (NADP)	Folate metabolism	XNR_3872 or XNR_5257
thiocoraline	16.51	PPDK	Pyruvate,phosphate dikinase	Glycolysis/Gluconeogenesis	XNR_4449
thiocoraline	14.43	GLNS	glutamine synthetase	Alanine, aspartate and glutamate metabolism	XNR_4631 or XNR_4658 or XNR_4684 or XNR_5219
thiocoraline	10.46	PRPPS	phosphoribosylpyrophosphate synthetase	Purine metabolism	XNR_2061
thiocoraline	9.38	ADSL1r	adenylsuccinate lyase	Purine metabolism	XNR_5550
thiocoraline	9.38	ADSS	adenylosuccinate synthase	Purine metabolism	XNR_3210
thiocoraline	8.99	ADSL2r	adenylosuccinate lyase	Purine metabolism	XNR_5550
thiocoraline	8.99	AIRCr	phosphoribosylaminoimidazole carboxylase	Purine metabolism	XNR_1959 and XNR_1960
thiocoraline	8.99	GARFT	phosphoribosylglycinamide formyltransferase	Purine metabolism	XNR_3869
thiocoraline	8.99	GLUPRT	glutamine phosphoribosyldiphosphate amidotransferase	Purine metabolism	XNR_2842
thiocoraline	8.99	PRAGSr	phosphoribosylglycinamide synthase	Purine metabolism	XNR_2857
thiocoraline	8.99	PRAIS	phosphoribosylaminoimidazole synthase	Purine metabolism	XNR_2841
thiocoraline	8.99	PRASCSi	phosphoribosylaminoimidazoles uccinocarboxamide synthase	Purine metabolism	XNR_2854
thiocoraline	8.99	PRFGS	phosphoribosylformylglycinamid ine synthase	Purine metabolism	XNR_2844 and XNR_2845 and XNR_2846
thiocoraline	8.73	AICART	phosphoribosylaminoimidazoleca rboxamide formyltransferase	Purine metabolism	XNR_3870
thiocoraline	8.73	IMPC	IMP cyclohydrolase	Purine metabolism	XNR_3870
thiocoraline	7.05	RPI	ribose-5-phosphate isomerase	Pentose phosphate pathway	XNR_4316

thiocoraline	7.01	RPE	ribulose 5-phosphate 3-epimerase	Pentose phosphate pathway	XNR_5386
thiocoraline	6.72	PGCD	phosphoglycerate dehydrogenase	Glycine, serine and threonine metabolism	XNR_1318
thiocoraline	6.72	PSERT	phosphoserine transaminase	Glycine, serine and threonine metabolism	XNR_2290
thiocoraline	6.72	PSP_L	phosphoserine phosphatase (L-serine)	Glycine, serine and threonine metabolism	XNR_5015
thiocoraline	4.58	ADSK	adenylyl-sulfate kinase	Cysteine metabolism	XNR_0713
thiocoraline	4.58	BPNT	3',5'-bisphosphate nucleotidase	Cysteine and methionine metabolism	XNR_1642
thiocoraline	4.58	SADT2	Sulfate adenyltransferase	Sulfur metabolism	XNR_0715 and XNR_0714
thiocoraline	4.58	SULR_syn	Hydrogen-sulfide:ferredoxin oxidoreductase	Sulfur metabolism	XNR_0710
thiocoraline	3.58	TKT2	transketolase	Pentose phosphate pathway	XNR_4929
thiocoraline	3.42	TALA	transaldolase	Pentose phosphate pathway	XNR_4928
thiocoraline	3.42	TKT1	transketolase	Pentose phosphate pathway	XNR_4929
thiocoraline	2.90	CYSS	cysteine synthase	Cysteine and methionine metabolism	XNR_0070 or XNR_1784
thiocoraline	1.96	NMNAT	nicotinamide-nucleotide adenylyltransferase	Nicotinate and nicotinamide metabolism	XNR_4358
thiocoraline	1.96	NMNHYD	Adenosine kinase	Nicotinate and nicotinamide metabolism	XNR_3310
thiocoraline	1.96	NNAM	nicotinamidase	Nicotinate and nicotinamide metabolism	XNR_1793
thiocoraline	1.96	NNDPR	nicotinate-nucleotide diphosphorylase (carboxylating)	Nicotinate and nicotinamide metabolism	XNR_3475
thiocoraline	1.96	NP1	Purine-nucleoside phosphorylase	Nicotinate and nicotinamide metabolism	XNR_4012
thiocoraline	1.96	NT5C	Nicotinate D-ribonucleotide phosphohydrolase	Nicotinate and nicotinamide metabolism	XNR_3310
thiocoraline	1.96	PNP	Purine-nucleoside phosphorylase	Nicotinate and nicotinamide metabolism	XNR_4012
thiocoraline	1.96	QULNS	quinolinate synthase	Nicotinate and nicotinamide metabolism	XNR_4717
thiocoraline	1.96	ASPO2	L-aspartate:NAD+ oxidoreductase (deaminating)	Nicotinate and nicotinamide metabolism	XNR_0361
thiocoraline	1.81	METAT	methionine adenosyltransferase	Methionine metabolism	XNR_5375
thiocoraline	1.68	SHSL2	O-succinylhomoserine lyase (H2S)	Cysteine and methionine metabolism	XNR_2948 or XNR_4037 or XNR_5540
thiocoraline	1.62	METS	methionine synthase	Methionine metabolism	XNR_5171
thiocoraline	1.61	MTHFR3_1	Methylenetetrahydrofolate reductase (NADPH)	Folate metabolism	XNR_4775

thiocoraline	0.97	HSDy	homoserine dehydrogenase (NADPH)	Glycine, serine and threonine metabolism	XNR_1479
thiocoraline	0.83	ANPRT	anthranilate phosphoribosyltransferase	Phenylalanine, tyrosine and tryptophan biosynthesis	XNR_4733
thiocoraline	0.83	IGPS	indole-3-glycerol-phosphate synthase	Phenylalanine, tyrosine and tryptophan biosynthesis	XNR_4840
thiocoraline	0.83	PRAIi	phosphoribosylanthranilate isomerase (irreversible)	Phenylalanine, tyrosine and tryptophan biosynthesis	XNR_4829
thiocoraline	0.83	TRPS1	tryptophan synthase (indoleglycerol phosphate)	Tryptophan metabolism	XNR_4842 and XNR_4841
thiocoraline	0.32	ASAD	aspartate-semialdehyde dehydrogenase	Threonine and Lysine metabolism	XNR_2758 or XNR_4304
thiocoraline	0.32	ASPK	aspartate kinase	Alanine, aspartate and glutamate metabolism	XNR_2759
thiocoraline	0.16	CHORS	chorismate synthase	Phenylalanine, tyrosine and tryptophan biosynthesis	XNR_5355
thiocoraline	0.16	DDPA	3-deoxy-D-arabino-heptulosonate 7-phosphate synthetase	Phenylalanine, tyrosine and tryptophan biosynthesis	XNR_0595 or XNR_4763
thiocoraline	0.16	DHQS	3-dehydroquinate synthase	Phenylalanine, tyrosine and tryptophan biosynthesis	XNR_5357
thiocoraline	0.16	DHQTi	3-dehydroquinate dehydratase, irreversible	Phenylalanine, tyrosine and tryptophan biosynthesis	XNR_4909
thiocoraline	0.16	PSCVT	3-phosphoshikimate 1-carboxyvinyltransferase	Phenylalanine, tyrosine and tryptophan biosynthesis	XNR_1588
thiocoraline	0.16	SHK3Dr	shikimate dehydrogenase	Phenylalanine, tyrosine and tryptophan biosynthesis	XNR_5354
thiocoraline	0.16	SHKK	shikimate kinase	Phenylalanine, tyrosine and tryptophan biosynthesis	XNR_5356

Table S4. Summary of all predicted FSEOF overexpression targets sorted by product of interest. Slopes indicate the flux of the associated target reaction per unit flux increase in the target reaction. Pathway and GPR information are displayed for each reaction.
