# Supplementary material for: Reconstruction of a Genome-Scale Metabolic Model of Streptomyces albus J1074: Improved Engineering Strategies in Natural Product Synthesis
Source: Metabolites. 2021 May 11;11(5):304. doi: 10.3390/metabo11050304 (PMC8150979; doi:10.3390/metabo11050304)
Supplement: Supplementary file 1 [file metabolites-11-00304-s001.zip › TableS2_new.pdf]

Reaction ID	Pathway	*Salb* -GEM genes	*Sco* -GEM genes
CYO2b	Oxidative phosphorylation	XNR_0617 and XNR_4724 and XNR_4726 and XNR_4729 and XNR_4930 and XNR_4933	(SCO1934 and SCO2156 and (SCO7234 or SCO2155) and SCO2151 and SCO1930 and SCO2154)
DXPS	Metabolism of cofactors and vitamins	XNR_0772	SCO6013 or SCO6768
DHDPS	Threonine and Lysine metabolism	XNR_1123	SCO1889 or SCO1912 or SCO5744 or SCO6292
MECDPDH2, MECDPH5_2	Terpenoid backbone biosynthesis	XNR_1162	SCO5696 or SCO6767
NH4t	Ionorganic ion transport and metabolism	XNR_1224	SCO5583 or SCO3085
KARA1, KARA1i, KARA2, KARI, KARI_23dhmb, KARI_23dhmp, KARI_3hmoa	Pantothenate and CoA biosynthesis; valine, leucine and isoleucine biosynthesis	XNR_1319	SCO5514 or SCO7154
HSDxi, HSDy	Glycine, serine and threonine metabolism	XNR_1479	SCO0420 or SCO5354
PSCVT	Phenylalanine, tyrosine and tryptophan biosynthesis	XNR_1588	SCO5212 or SCO6819
PRPPS	Purine metabolism	XNR_2061	SCO0782 or SCO3123
ATNS_nh4	Phenylalanine, tyrosine and tryptophan biosynthesis	XNR_3034 and XNR_4836	(SCO3213 and SCO3214) or (SCO3213 and SCO2043)
APCS, SPMS, SPRMS	Glutathione metabolism; cysteine and methionine metabolism	XNR_3191	SCO3655 or SCO2455
DHAD1, DHAD2	Valine, leucine and isoleucine biosynthesis	XNR_3504	SCO1176 or SCO1888 or SCO3345
HMBS	Porphyrin and chlorophyll metabolism	XNR_3532	SCO7343 or SCO3318
AMMQLT9, AMMQT9	Ubiquinone and other terpenoid-quinone biosynthesis	XNR_3626	SCO4556 or SCO5940
3OAS100, 3OAS110, 3OAS120, 3OAS121, 3OAS130, 3OAS140, 3OAS141, 3OAS150, 3OAS160, 3OAS161, 3OAS170, 3OAS180, 3OAS181, 3OAS50, 3OAS60, 3OAS70, 3OAS80, 3OAS90, 3OASai110, 3OASai130, 3OASai150, 3OASai170, 3OASai70, 3OASai90, 3OASi100, 3OASi110, 3OASi120, 3OASi130, 3OASi140, 3OASi150, 3OASi160, 3OASi170, 3OASi180, 3OASi60, 3OASi70, 3OASi80, 3OASi90, CDAS6, KAS14, OGMEACPS2, OPMEACPS	Fatty acid biosynthesis; biotin metabolism	XNR_4509	SCO0548 or SCO2390 or SCO1266 or SCO3248
MCOATA	Fatty acid biosynthesis	XNR_4510 and XNR_4512	(SCO2387 and (SCO2389 or SCO0549 or SCO1267 or SCO1272))
CYO1ab, CYO2a	Oxidative phosphorylation	XNR_4730 and XNR_4731 and XNR_4732	(SCO2150 and SCO2149 and (SCO7236 or SCO2148 or SCO7120))
ANPRT	Phenylalanine, tyrosine and tryptophan biosynthesis	XNR_4733	SCO2147 or SCO3212
HOPNTAL3	Benzoate degradation	XNR_4831	SCO2048 or SCP1301 or SCP153c
IGPS	Phenylalanine, tyrosine and tryptophan	XNR_4840	SCO2039 or SCO3211
CHORM	Phenylalanine, tyrosine and tryptophan biosynthesis	XNR_4859	SCO1762 or SCO2019 or SCO4784
PPND	Phenylalanine, tyrosine and tryptophan	XNR_5061	SCO1761 or SCO3221
PC6YM	Porphyrin and chlorophyll metabolism	XNR_5293	SCO1555 or SCO1856
GTPCII2	Metabolism of cofactors and vitamins	XNR_5408	SCO1441 or SCO2687 or SCO6655

Table S2. Metabolic reactions in *Salb* -GEM and *Sco* -GEM associated with essential genes predicted in *Salb* -GEM with multiple paralogues in *Sco* -GEM. The table is organized by *S. albus* genes in ascending order.
