# Supplementary material for: Reconstruction of a Genome-Scale Metabolic Model of Streptomyces albus J1074: Improved Engineering Strategies in Natural Product Synthesis
Source: Metabolites. 2021 May 11;11(5):304. doi: 10.3390/metabo11050304 (PMC8150979; doi:10.3390/metabo11050304)
Supplement: Supplementary file 1 [file metabolites-11-00304-s001.zip › TableS3_new.pdf]

**Reaction ID**	**Pathway**	***Salb* -GEM genes**	***Sco* -GEM genes**
CPMPS	Porphyrin and chlorophyll metabolism	(XNR_0124 and XNR_2127) or (XNR_4999 and XNR_2127)	SCO1821 and SCO3180
PC17M	Porphyrin and chlorophyll metabolism	XNR_4670 or XNR_4968	SCO1857
REDS32	Undecylprodigiosin Biosynthesis	(XNR_4512 and XNR_0019) or (XNR_4512 and XNR_5920)	SCO2387 and SCO5890
MMSAD1, MMSAD2, MMSAD3, MMSAD5, MMTSAO	Valine, leucine and isoleucine degradation, propanoate metabolism	XNR_0154 or XNR_4241	SCO2726
GCALDD	Glyoxylate and dicarboxylate metabolism	XNR_0363 or XNR_1178 or XNR_5220	SCO3486
MTHFC, MTHFD	Folate metabolism	XNR_3872 or XNR_5257	SCO4824
MPTS	Folate biosynthesis	XNR_0122 or XNR_1602	SCO5201
MOADSUx	Metabolism of cofactors and vitamins	(XNR_1347 and XNR_0122) or (XNR_1347 and XNR_1602)	SCO5486 and SCO5201
PSSA120, PSSA140, PSSA141, PSSA150, PSSA160, PSSA161, PSSA170, PSSA180, PSSA181, PSSAai150, PSSAai170, PSSAi140, PSSAi150, PSSAi160, PSSAi170, PSSAi180	Glycerophospholipid metabolism	XNR_0462 or XNR_3542	SCO6467

Table S3. Metabolic reactions in *Salb* -GEM and *Sco* -GEM associated with essential genes predicted in *Sco* -GEM with multiple paralogues in *Salb* -GEM. The table is organized by *S. coelicolor* genes in ascending order.
